# Supplementary material for: Modeling glioblastoma heterogeneity as a dynamic network of cell states
Source: Mol Syst Biol. 2021 Sep 16;17(9):e10105. doi: 10.15252/msb.202010105 (PMC8444284; doi:10.15252/msb.202010105)
Supplement: Supplementary file 6 — Source Data for Figure 5 [file MSB-17-e10105-s004.zip › Figure5A_sourcedata/GSEA_3017/nosigdb_stateE.GseaPreranked.1622795031093/RICHARDS_NOWAKOWSKI_SCIENCE_2017_MGE_IPC1_UPREG.html]

Details for gene set RICHARDS\_NOWAKOWSKI\_SCIENCE\_2017\_MGE\_IPC1\_UPREG[GSEA]

|  || Dataset | state63017 |
| Phenotype | NoPhenotypeAvailable |
| Upregulated in class | na\_neg |
| GeneSet | RICHARDS\_NOWAKOWSKI\_SCIENCE\_2017\_MGE\_IPC1\_UPREG |
| Enrichment Score (ES) | -0.5927372 |
| Normalized Enrichment Score (NES) | -3.493034 |
| Nominal p-value | 0.0 |
| FDR q-value | 0.0 |
| FWER p-Value | 0.0 |
Table: GSEA Results Summary

  

Fig 1: Enrichment plot: RICHARDS\_NOWAKOWSKI\_SCIENCE\_2017\_MGE\_IPC1\_UPREG      
 Profile of the Running ES Score & Positions of GeneSet Members on the Rank Ordered List

  

| PROBE | GENE SYMBOL | GENE\_TITLE | RANK IN GENE LIST | RANK METRIC SCORE | RUNNING ES | CORE ENRICHMENT || 1 | FRMD4B |  |  | 48 | 0.532 | -0.0669 | No |
| 2 | HSPA5 |  |  | 73 | 0.449 | -0.0992 | No |
| 3 | NES |  |  | 92 | 0.414 | -0.1229 | No |
| 4 | TMSB4X |  |  | 93 | 0.412 | -0.1201 | No |
| 5 | CD99 |  |  | 107 | 0.389 | -0.1366 | No |
| 6 | LGALS1 |  |  | 113 | 0.368 | -0.1415 | No |
| 7 | MBNL2 |  |  | 173 | 0.316 | -0.2260 | No |
| 8 | NLGN4X |  |  | 177 | 0.312 | -0.2284 | No |
| 9 | CALM2 |  |  | 191 | 0.303 | -0.2454 | No |
| 10 | GNG11 |  |  | 210 | 0.289 | -0.2699 | No |
| 11 | CDK6 |  |  | 238 | 0.269 | -0.3078 | No |
| 12 | QKI |  |  | 241 | 0.267 | -0.3089 | No |
| 13 | HMGCR |  |  | 251 | 0.261 | -0.3204 | No |
| 14 | WWTR1 |  |  | 263 | 0.256 | -0.3349 | No |
| 15 | LIN54 |  |  | 271 | -0.250 | -0.3435 | No |
| 16 | ARHGAP11B |  |  | 277 | -0.252 | -0.3491 | No |
| 17 | TUBA1C |  |  | 282 | -0.253 | -0.3533 | No |
| 18 | ZMYM1 |  |  | 283 | -0.253 | -0.3516 | No |
| 19 | NUP37 |  |  | 286 | -0.255 | -0.3529 | No |
| 20 | ZFP36L1 |  |  | 287 | -0.255 | -0.3512 | No |
| 21 | ZNF850 |  |  | 291 | -0.257 | -0.3539 | No |
| 22 | RAN |  |  | 298 | -0.257 | -0.3610 | No |
| 23 | CCDC77 |  |  | 306 | -0.259 | -0.3695 | No |
| 24 | AHI1 |  |  | 310 | -0.261 | -0.3722 | No |
| 25 | AHCY |  |  | 317 | -0.262 | -0.3792 | No |
| 26 | TGIF1 |  |  | 318 | -0.263 | -0.3775 | No |
| 27 | TUBG1 |  |  | 323 | -0.263 | -0.3816 | No |
| 28 | NUDCD2 |  |  | 330 | -0.264 | -0.3887 | No |
| 29 | BRD8 |  |  | 341 | -0.266 | -0.4016 | No |
| 30 | ARHGEF39 |  |  | 346 | -0.268 | -0.4057 | No |
| 31 | RPL22L1 |  |  | 350 | -0.269 | -0.4083 | No |
| 32 | KIAA0586 |  |  | 362 | -0.272 | -0.4226 | No |
| 33 | DLEU2 |  |  | 365 | -0.272 | -0.4237 | No |
| 34 | APOLD1 |  |  | 374 | -0.275 | -0.4336 | No |
| 35 | CEP44 |  |  | 377 | -0.275 | -0.4347 | No |
| 36 | TBC1D31 |  |  | 380 | -0.276 | -0.4358 | No |
| 37 | HNRNPA2B1 |  |  | 391 | -0.279 | -0.4487 | No |
| 38 | CCAR1 |  |  | 396 | -0.282 | -0.4527 | No |
| 39 | SNRPG |  |  | 398 | -0.283 | -0.4522 | No |
| 40 | GDI2 |  |  | 401 | -0.283 | -0.4533 | No |
| 41 | ARHGAP19 |  |  | 414 | -0.287 | -0.4690 | No |
| 42 | NIF3L1 |  |  | 418 | -0.289 | -0.4715 | No |
| 43 | SCLT1 |  |  | 419 | -0.289 | -0.4695 | No |
| 44 | MYBL1 |  |  | 432 | -0.294 | -0.4852 | No |
| 45 | LSM8 |  |  | 441 | -0.297 | -0.4950 | No |
| 46 | ZNF43 |  |  | 449 | -0.299 | -0.5033 | No |
| 47 | CEP57L1 |  |  | 450 | -0.299 | -0.5013 | No |
| 48 | CDC7 |  |  | 451 | -0.299 | -0.4993 | No |
| 49 | PNRC2 |  |  | 461 | -0.304 | -0.5105 | No |
| 50 | CNTRL |  |  | 462 | -0.304 | -0.5084 | No |
| 51 | BCHE |  |  | 471 | -0.305 | -0.5181 | No |
| 52 | NEIL3 |  |  | 475 | -0.307 | -0.5205 | No |
| 53 | EXOSC9 |  |  | 481 | -0.308 | -0.5258 | No |
| 54 | RSRC1 |  |  | 494 | -0.312 | -0.5413 | No |
| 55 | NUP205 |  |  | 495 | -0.313 | -0.5393 | No |
| 56 | ILF2 |  |  | 497 | -0.313 | -0.5386 | No |
| 57 | CEP97 |  |  | 499 | -0.313 | -0.5380 | No |
| 58 | COX17 |  |  | 505 | -0.316 | -0.5433 | No |
| 59 | PIK3R3 |  |  | 508 | -0.317 | -0.5441 | No |
| 60 | SRRT |  |  | 509 | -0.317 | -0.5420 | No |
| 61 | BARD1 |  |  | 510 | -0.318 | -0.5398 | No |
| 62 | DNA2 |  |  | 513 | -0.318 | -0.5407 | No |
| 63 | CCDC150 |  |  | 516 | -0.319 | -0.5415 | No |
| 64 | SAPCD2 |  |  | 517 | -0.319 | -0.5393 | No |
| 65 | KIF20A |  |  | 523 | -0.321 | -0.5445 | No |
| 66 | RHEB |  |  | 528 | -0.323 | -0.5483 | No |
| 67 | NUP62 |  |  | 529 | -0.323 | -0.5461 | No |
| 68 | CEP192 |  |  | 531 | -0.324 | -0.5454 | No |
| 69 | CEP135 |  |  | 532 | -0.325 | -0.5433 | No |
| 70 | ACTL6A |  |  | 533 | -0.325 | -0.5411 | No |
| 71 | TPRKB |  |  | 540 | -0.329 | -0.5477 | No |
| 72 | ING1 |  |  | 541 | -0.329 | -0.5455 | No |
| 73 | H3F3A |  |  | 542 | -0.329 | -0.5433 | No |
| 74 | JADE1 |  |  | 543 | -0.329 | -0.5411 | No |
| 75 | CDC25C |  |  | 549 | -0.332 | -0.5462 | No |
| 76 | GPSM2 |  |  | 550 | -0.334 | -0.5440 | No |
| 77 | PLK4 |  |  | 551 | -0.334 | -0.5418 | No |
| 78 | C2orf69 |  |  | 556 | -0.338 | -0.5454 | No |
| 79 | SAP30 |  |  | 558 | -0.339 | -0.5446 | No |
| 80 | FAM83D |  |  | 559 | -0.340 | -0.5424 | No |
| 81 | TROAP |  |  | 562 | -0.341 | -0.5430 | No |
| 82 | LSM5 |  |  | 568 | -0.342 | -0.5481 | No |
| 83 | HMGB3 |  |  | 577 | -0.348 | -0.5575 | No |
| 84 | RCC1 |  |  | 582 | -0.350 | -0.5611 | No |
| 85 | GEN1 |  |  | 584 | -0.351 | -0.5602 | No |
| 86 | NUP107 |  |  | 586 | -0.352 | -0.5593 | No |
| 87 | DDX39A |  |  | 590 | -0.353 | -0.5614 | No |
| 88 | CDK5RAP2 |  |  | 591 | -0.353 | -0.5590 | No |
| 89 | CENPC |  |  | 596 | -0.356 | -0.5625 | No |
| 90 | ASRGL1 |  |  | 608 | -0.362 | -0.5763 | No |
| 91 | KIF18B |  |  | 612 | -0.363 | -0.5782 | No |
| 92 | BTG3 |  |  | 615 | -0.365 | -0.5788 | No |
| 93 | RFWD3 |  |  | 617 | -0.365 | -0.5778 | No |
| 94 | C18orf54 |  |  | 619 | -0.366 | -0.5768 | No |
| 95 | G2E3 |  |  | 624 | -0.371 | -0.5802 | No |
| 96 | LCORL |  |  | 627 | -0.374 | -0.5807 | No |
| 97 | NEK2 |  |  | 628 | -0.374 | -0.5782 | No |
| 98 | EXOSC8 |  |  | 630 | -0.376 | -0.5771 | No |
| 99 | CDKN2D |  |  | 638 | -0.381 | -0.5849 | No |
| 100 | MPHOSPH9 |  |  | 644 | -0.384 | -0.5896 | No |
| 101 | TTF2 |  |  | 646 | -0.386 | -0.5885 | No |
| 102 | GAS2L3 |  |  | 647 | -0.388 | -0.5860 | No |
| 103 | PHF19 |  |  | 648 | -0.391 | -0.5833 | No |
| 104 | ZEB1 |  |  | 653 | -0.396 | -0.5866 | No |
| 105 | SHCBP1 |  |  | 655 | -0.398 | -0.5854 | No |
| 106 | SEPHS1 |  |  | 661 | -0.401 | -0.5901 | Yes |
| 107 | PBX3 |  |  | 662 | -0.403 | -0.5874 | Yes |
| 108 | RHNO1 |  |  | 664 | -0.406 | -0.5861 | Yes |
| 109 | MNS1 |  |  | 666 | -0.408 | -0.5849 | Yes |
| 110 | SAE1 |  |  | 668 | -0.408 | -0.5836 | Yes |
| 111 | KIF18A |  |  | 673 | -0.412 | -0.5868 | Yes |
| 112 | NUP50 |  |  | 677 | -0.415 | -0.5884 | Yes |
| 113 | CIT |  |  | 680 | -0.418 | -0.5885 | Yes |
| 114 | RBMX |  |  | 682 | -0.421 | -0.5872 | Yes |
| 115 | H2AFV |  |  | 684 | -0.423 | -0.5859 | Yes |
| 116 | CENPO |  |  | 686 | -0.424 | -0.5845 | Yes |
| 117 | STIL |  |  | 692 | -0.425 | -0.5890 | Yes |
| 118 | CKAP5 |  |  | 694 | -0.426 | -0.5876 | Yes |
| 119 | DIAPH3 |  |  | 696 | -0.427 | -0.5863 | Yes |
| 120 | RANGAP1 |  |  | 698 | -0.429 | -0.5849 | Yes |
| 121 | ANP32B |  |  | 699 | -0.431 | -0.5820 | Yes |
| 122 | CENPL |  |  | 703 | -0.432 | -0.5835 | Yes |
| 123 | PARPBP |  |  | 705 | -0.434 | -0.5821 | Yes |
| 124 | TEX30 |  |  | 709 | -0.436 | -0.5836 | Yes |
| 125 | MZT1 |  |  | 711 | -0.437 | -0.5822 | Yes |
| 126 | LMNB2 |  |  | 713 | -0.438 | -0.5807 | Yes |
| 127 | SKA1 |  |  | 719 | -0.447 | -0.5851 | Yes |
| 128 | TTK |  |  | 724 | -0.454 | -0.5879 | Yes |
| 129 | POLQ |  |  | 726 | -0.455 | -0.5863 | Yes |
| 130 | RAD21 |  |  | 728 | -0.458 | -0.5848 | Yes |
| 131 | SPAG5 |  |  | 729 | -0.458 | -0.5817 | Yes |
| 132 | CCNF |  |  | 732 | -0.460 | -0.5816 | Yes |
| 133 | LBR |  |  | 739 | -0.463 | -0.5873 | Yes |
| 134 | MGME1 |  |  | 742 | -0.468 | -0.5871 | Yes |
| 135 | SMC3 |  |  | 743 | -0.470 | -0.5840 | Yes |
| 136 | NUCKS1 |  |  | 744 | -0.471 | -0.5808 | Yes |
| 137 | PTMA |  |  | 745 | -0.475 | -0.5777 | Yes |
| 138 | CHD7 |  |  | 746 | -0.480 | -0.5745 | Yes |
| 139 | MIS18A |  |  | 750 | -0.483 | -0.5757 | Yes |
| 140 | KNSTRN |  |  | 751 | -0.485 | -0.5724 | Yes |
| 141 | PRR11 |  |  | 755 | -0.488 | -0.5736 | Yes |
| 142 | SPDL1 |  |  | 758 | -0.491 | -0.5732 | Yes |
| 143 | CDCA3 |  |  | 759 | -0.497 | -0.5699 | Yes |
| 144 | DCP2 |  |  | 762 | -0.500 | -0.5695 | Yes |
| 145 | MXD3 |  |  | 763 | -0.502 | -0.5662 | Yes |
| 146 | GMNN |  |  | 766 | -0.510 | -0.5657 | Yes |
| 147 | RTKN2 |  |  | 767 | -0.511 | -0.5623 | Yes |
| 148 | LRR1 |  |  | 768 | -0.512 | -0.5589 | Yes |
| 149 | KIF5B |  |  | 770 | -0.515 | -0.5569 | Yes |
| 150 | DTYMK |  |  | 774 | -0.522 | -0.5579 | Yes |
| 151 | DEPDC1B |  |  | 779 | -0.533 | -0.5602 | Yes |
| 152 | GLCCI1 |  |  | 780 | -0.535 | -0.5566 | Yes |
| 153 | CENPA |  |  | 782 | -0.538 | -0.5545 | Yes |
| 154 | SKA3 |  |  | 783 | -0.539 | -0.5509 | Yes |
| 155 | NCAPD2 |  |  | 784 | -0.540 | -0.5473 | Yes |
| 156 | CEP152 |  |  | 785 | -0.542 | -0.5437 | Yes |
| 157 | FANCD2 |  |  | 787 | -0.544 | -0.5415 | Yes |
| 158 | INCENP |  |  | 789 | -0.558 | -0.5393 | Yes |
| 159 | HAUS8 |  |  | 792 | -0.560 | -0.5385 | Yes |
| 160 | CDC20 |  |  | 793 | -0.562 | -0.5347 | Yes |
| 161 | HMGN2 |  |  | 795 | -0.564 | -0.5324 | Yes |
| 162 | RACGAP1 |  |  | 796 | -0.566 | -0.5287 | Yes |
| 163 | HMGB1 |  |  | 797 | -0.567 | -0.5249 | Yes |
| 164 | CENPJ |  |  | 799 | -0.568 | -0.5226 | Yes |
| 165 | HES6 |  |  | 800 | -0.569 | -0.5188 | Yes |
| 166 | TUBB |  |  | 802 | -0.570 | -0.5164 | Yes |
| 167 | TUBB4B |  |  | 804 | -0.573 | -0.5141 | Yes |
| 168 | CENPN |  |  | 805 | -0.574 | -0.5103 | Yes |
| 169 | USP1 |  |  | 806 | -0.575 | -0.5064 | Yes |
| 170 | CDCA8 |  |  | 808 | -0.585 | -0.5040 | Yes |
| 171 | PLK1 |  |  | 809 | -0.586 | -0.5001 | Yes |
| 172 | DEK |  |  | 812 | -0.591 | -0.4991 | Yes |
| 173 | FOXM1 |  |  | 814 | -0.593 | -0.4966 | Yes |
| 174 | DEPDC1 |  |  | 815 | -0.596 | -0.4926 | Yes |
| 175 | CDCA2 |  |  | 816 | -0.597 | -0.4887 | Yes |
| 176 | LMNB1 |  |  | 818 | -0.606 | -0.4861 | Yes |
| 177 | MND1 |  |  | 821 | -0.615 | -0.4849 | Yes |
| 178 | PIF1 |  |  | 822 | -0.616 | -0.4808 | Yes |
| 179 | AURKA |  |  | 824 | -0.623 | -0.4781 | Yes |
| 180 | CKAP2L |  |  | 826 | -0.625 | -0.4754 | Yes |
| 181 | CCDC34 |  |  | 827 | -0.626 | -0.4713 | Yes |
| 182 | AURKB |  |  | 829 | -0.634 | -0.4685 | Yes |
| 183 | BUB3 |  |  | 834 | -0.641 | -0.4701 | Yes |
| 184 | CENPW |  |  | 835 | -0.643 | -0.4658 | Yes |
| 185 | CDKN3 |  |  | 836 | -0.653 | -0.4615 | Yes |
| 186 | BUB1 |  |  | 837 | -0.653 | -0.4571 | Yes |
| 187 | ITGB3BP |  |  | 838 | -0.661 | -0.4527 | Yes |
| 188 | ANP32E |  |  | 841 | -0.676 | -0.4511 | Yes |
| 189 | KIF22 |  |  | 842 | -0.678 | -0.4466 | Yes |
| 190 | BIRC5 |  |  | 843 | -0.679 | -0.4421 | Yes |
| 191 | NCAPH |  |  | 845 | -0.685 | -0.4390 | Yes |
| 192 | ZWINT |  |  | 847 | -0.692 | -0.4358 | Yes |
| 193 | SPC24 |  |  | 848 | -0.694 | -0.4312 | Yes |
| 194 | BRCA2 |  |  | 850 | -0.694 | -0.4281 | Yes |
| 195 | VRK1 |  |  | 852 | -0.711 | -0.4248 | Yes |
| 196 | ECT2 |  |  | 854 | -0.717 | -0.4215 | Yes |
| 197 | KIF2C |  |  | 855 | -0.721 | -0.4167 | Yes |
| 198 | NUF2 |  |  | 856 | -0.721 | -0.4119 | Yes |
| 199 | CENPK |  |  | 857 | -0.722 | -0.4070 | Yes |
| 200 | FAM111A |  |  | 858 | -0.727 | -0.4022 | Yes |
| 201 | ANLN |  |  | 860 | -0.733 | -0.3988 | Yes |
| 202 | KIF4A |  |  | 861 | -0.736 | -0.3939 | Yes |
| 203 | HMMR |  |  | 863 | -0.740 | -0.3904 | Yes |
| 204 | FBXO5 |  |  | 864 | -0.742 | -0.3855 | Yes |
| 205 | CCNB2 |  |  | 865 | -0.745 | -0.3805 | Yes |
| 206 | ARHGAP11A |  |  | 867 | -0.756 | -0.3769 | Yes |
| 207 | NDC80 |  |  | 869 | -0.762 | -0.3733 | Yes |
| 208 | ORC6 |  |  | 870 | -0.766 | -0.3682 | Yes |
| 209 | BUB1B |  |  | 871 | -0.766 | -0.3631 | Yes |
| 210 | CCNA2 |  |  | 872 | -0.770 | -0.3580 | Yes |
| 211 | KIF23 |  |  | 873 | -0.772 | -0.3528 | Yes |
| 212 | MELK |  |  | 874 | -0.776 | -0.3476 | Yes |
| 213 | TMPO |  |  | 875 | -0.778 | -0.3425 | Yes |
| 214 | KIF20B |  |  | 876 | -0.779 | -0.3373 | Yes |
| 215 | CEP55 |  |  | 877 | -0.780 | -0.3321 | Yes |
| 216 | CKAP2 |  |  | 878 | -0.781 | -0.3269 | Yes |
| 217 | NCAPG2 |  |  | 879 | -0.781 | -0.3216 | Yes |
| 218 | PTTG1 |  |  | 880 | -0.791 | -0.3164 | Yes |
| 219 | H2AFX |  |  | 882 | -0.799 | -0.3125 | Yes |
| 220 | KIFC1 |  |  | 883 | -0.807 | -0.3071 | Yes |
| 221 | KIF14 |  |  | 885 | -0.809 | -0.3032 | Yes |
| 222 | EZH2 |  |  | 886 | -0.810 | -0.2978 | Yes |
| 223 | UBE2S |  |  | 887 | -0.815 | -0.2924 | Yes |
| 224 | KIF15 |  |  | 890 | -0.834 | -0.2897 | Yes |
| 225 | CDCA5 |  |  | 892 | -0.841 | -0.2856 | Yes |
| 226 | H2AFZ |  |  | 893 | -0.849 | -0.2799 | Yes |
| 227 | RAD51AP1 |  |  | 894 | -0.853 | -0.2743 | Yes |
| 228 | KPNA2 |  |  | 895 | -0.858 | -0.2685 | Yes |
| 229 | DBF4 |  |  | 896 | -0.864 | -0.2628 | Yes |
| 230 | SMC2 |  |  | 897 | -0.867 | -0.2570 | Yes |
| 231 | CENPE |  |  | 899 | -0.873 | -0.2526 | Yes |
| 232 | PBK |  |  | 900 | -0.880 | -0.2468 | Yes |
| 233 | SMC4 |  |  | 902 | -0.886 | -0.2423 | Yes |
| 234 | MIS18BP1 |  |  | 904 | -0.889 | -0.2379 | Yes |
| 235 | C21orf58 |  |  | 905 | -0.893 | -0.2319 | Yes |
| 236 | CCNB1 |  |  | 906 | -0.895 | -0.2260 | Yes |
| 237 | CKS1B |  |  | 909 | -0.912 | -0.2228 | Yes |
| 238 | HJURP |  |  | 910 | -0.921 | -0.2167 | Yes |
| 239 | CKS2 |  |  | 911 | -0.922 | -0.2105 | Yes |
| 240 | GTSE1 |  |  | 912 | -0.941 | -0.2043 | Yes |
| 241 | KIF11 |  |  | 913 | -0.957 | -0.1979 | Yes |
| 242 | TYMS |  |  | 917 | -0.965 | -0.1958 | Yes |
| 243 | CENPU |  |  | 918 | -0.971 | -0.1894 | Yes |
| 244 | PRC1 |  |  | 919 | -0.980 | -0.1828 | Yes |
| 245 | MAD2L1 |  |  | 921 | -0.990 | -0.1777 | Yes |
| 246 | UBE2T |  |  | 922 | -0.994 | -0.1711 | Yes |
| 247 | DLGAP5 |  |  | 923 | -0.998 | -0.1644 | Yes |
| 248 | SPC25 |  |  | 924 | -1.033 | -0.1575 | Yes |
| 249 | HIST1H1E |  |  | 925 | -1.049 | -0.1505 | Yes |
| 250 | ESCO2 |  |  | 926 | -1.067 | -0.1434 | Yes |
| 251 | UBE2C |  |  | 928 | -1.108 | -0.1375 | Yes |
| 252 | NUSAP1 |  |  | 929 | -1.112 | -0.1301 | Yes |
| 253 | TPX2 |  |  | 930 | -1.124 | -0.1226 | Yes |
| 254 | NCAPG |  |  | 931 | -1.142 | -0.1150 | Yes |
| 255 | ASPM |  |  | 932 | -1.146 | -0.1073 | Yes |
| 256 | ATAD2 |  |  | 933 | -1.162 | -0.0996 | Yes |
| 257 | CENPF |  |  | 935 | -1.200 | -0.0931 | Yes |
| 258 | HIST1H1C |  |  | 936 | -1.220 | -0.0849 | Yes |
| 259 | HMGB2 |  |  | 937 | -1.238 | -0.0767 | Yes |
| 260 | CDK1 |  |  | 938 | -1.248 | -0.0684 | Yes |
| 261 | RRM2 |  |  | 940 | -1.403 | -0.0605 | Yes |
| 262 | MKI67 |  |  | 941 | -1.407 | -0.0511 | Yes |
| 263 | TOP2A |  |  | 942 | -1.417 | -0.0416 | Yes |
| 264 | HIST1H1D |  |  | 944 | -1.810 | -0.0310 | Yes |
| 265 | HIST1H1B |  |  | 945 | -1.903 | -0.0184 | Yes |
| 266 | HIST1H4C |  |  | 946 | -2.752 | -0.0000 | Yes |
Table: GSEA details [plain text format]

  

Fig 2: RICHARDS\_NOWAKOWSKI\_SCIENCE\_2017\_MGE\_IPC1\_UPREG: Random ES distribution      
 Gene set null distribution of ES for **RICHARDS\_NOWAKOWSKI\_SCIENCE\_2017\_MGE\_IPC1\_UPREG**
